# Multifaceted Signaling Networks Mediated by Abscisic Acid Insensitive 4

**DOI:** 10.1016/j.xplc.2020.100040

**Published:** 2020-03-07

**Authors:** Umashankar Chandrasekaran, Xiaofeng Luo, Wenguan Zhou, Kai Shu

**Affiliations:** 1 School of Ecology and Environment, Northwestern Polytechnical University, Xi'an 710012, China; 2 Institute of Ecological Agriculture, Sichuan Agricultural University, Chengdu 611130, China

**Keywords:** ABI4, transcription factor, target genes, phytohormones, crosstalk

## Abstract

Although ABSCISIC ACID INSENSITIVE 4 (ABI4) was initially demonstrated as a key positive regulator in the phytohormone abscisic acid (ABA) signaling cascade, multiple studies have now shown that it is actually involved in the regulation of several other cascades, including diverse phytohormone biogenesis and signaling pathways, various developmental processes (such as seed dormancy and germination, seedling establishment, and root development), disease resistance and lipid metabolism. Consistent with its versatile biological functions, ABI4 either activates or represses transcription of its target genes. The upstream regulators of ABI4 at both the transcription and post-transcription levels have also been documented in recent years. Consequently, a complicated network consisting of the direct target genes and upstream regulators of ABI4, through which ABI4 participates in several phytohormone crosstalk networks, has been generated. In this review, we summarize current understanding of the sophisticated ABI4-mediated molecular networks, mainly focusing on diverse phytohormone (including ABA, gibberellin, cytokinin, ethylene, auxin, and jasmonic acid) crosstalks. We also discuss the potential mechanisms through which ABI4 receives the ABA signal, focusing on protein phosphorylation modification events.

## Teaser

In this review, the versatile functions of transcription factor ABI4 in diverse phytohormone-mediated crosstalk networks, its distinct roles in transcriptional regulation as an activator or repressor, controversial viewpoints about the role of ABI4 in plastid retrograde signaling, and unidentified protein phosphorylation events are summarized and discussed.

## Introduction

The phytohormone abscisic acid (ABA) regulates many aspects of plant growth and development, including embryogenesis, seed dormancy and germination, plant–water relations, and tolerance to a variety of abiotic environmental stresses (Bulgakov et al., 2019). ABA signaling in plants requires ABA sensing by the core signaling components, which consist of several signal transducers and transcription factors such as the ABA receptors pyrabactin resistance1 (PYR)/pyrabactin resistance1-like (PYL)/regulatory components of ABA receptor (RCAR), the type 2C protein phosphatases ABI1 and ABI2 (PP2Cs), SUCROSE NONFERMENTING 1-related protein kinases 2 (Soon et al., 2012), downstream B3 transcription factor ABI3, AP2 transcription factor ABI4, and bZIP transcription factor ABI5 (Cutler et al., 2010, Umezawa et al., 2010).

Among these, PYR/PYL/RCAR proteins directly bind to ABA and inhibit the activity of PP2Cs (Joshi-Saha et al., 2011, Gupta et al., 2019, Vaidya et al., 2019), leading to the derepression of SnRK2 protein kinases and the activation of downstream genes, such as bZIP transcription factor ABI5; in the case of the AP2 domain-containing transcription factor Abscisic Acid Insensitive 4 (ABI4), only indirect transcriptional regulation by SnRK2 kinases via RAV1 has been reported, and the protein kinases responsible for its direct phosphorylation have remained elusive so far (Cutler et al., 2010, Umezawa et al., 2010, Feng et al., 2014). Initially, *abi4* was identified as one of five ABA-insensitive mutants (Finkelstein, 1994) and later as a salt- and mannitol-insensitive mutant (Quesada et al., 2000). Interestingly, ABI4 also possesses several other names, including Sucrose-Uncoupled 6 (Huijser et al., 2000), Sugar-Insensitive 5 (Laby et al., 2000), Impaired Sucrose Induction 3 (Rook et al., 2002), Glucose Insensitive 6 (GIN6) (Arenas-Huertero et al., 2000), and SALOBRENO 5 (SAN5) (Quesada et al., 2000). These reports indicate that ABI4 has versatile roles in several other regulatory pathways beyond ABA.

ABI4 is a member of the AP2/ERF family, and members of this family bind specifically to the ABRE (Mizoi et al., 2012), CE1 (Niu et al., 2002), S-box (Acevedo-Hernández et al., 2005), G-box (Koussevitzky et al., 2007), CCAC motif (Rook et al., 2006, Koussevitzky et al., 2007, Shu et al., 2013, Shu et al., 2016a), and CACCG elements (Hu et al., 2012) in the promoters of stress-responsive genes and regulate their expression. Sequence alignment of ABI4-related proteins from diverse species showed conservation of the characteristic AP2/ERF domain in the N-terminal region, in which a glutamic acid residue at the 69th position is essential for the function of ABI4 (Laby et al., 2000, Wind et al., 2013, Gregorio et al., 2014). Despite the similarity between the AP2/DNA-binding domains of ABI4 and other members of the DREBA subgroup, ABI4 stands out as a unique member in the A3 clade based on its sequence (Dietz et al., 2010). Orthologs of ABI4 have been reported in many other plant species, including maize (Niu et al., 2002) and rice (Wang et al., 2015), and also in some aquatic plants such as lotus (*Nelumbo nucifera*) (Ming et al., 2013), indicating that this factor is conserved in most land as well as aquatic plants (Wind et al., 2013, Gregorio et al., 2014).

Over the decades, ABI4 has emerged as a central player in some phytohormone signaling processes during plant development and biotic/abiotic stress responses. How environmental cues are perceived and integrated into alterations of the activity of the ABI4 transcription factor is still largely a conundrum. In this updated review, we present the recent evidence for the mechanisms by which ABI4 modulates various plant signaling modules beyond the ABA signaling cascade.

## ABI4 Acts as Both a Gene Expression Activator and Repressor

Genetic studies, especially in the model plant *Arabidopsis*, have identified a large number of loci involved in ABA response. These loci likely act in multiple overlapping response pathways (Nakamura et al., 2001, Chen et al., 2020). ABI3, ABI4, and ABI5 are three well-characterized positive regulators of ABA signaling among many. ABI4 activates or represses gene expression by binding to specific DNA fragments in promoters via its AP2 domain (Wind et al., 2013).

Previous studies showed that ABI4 mediates ABA- and sugar-induced repression of photosynthetically dependent nuclear genes, and this repression is correlated with ABI4 binding to a designated motif, the S-box (CACYKSCA) (Acevedo-Hernández et al., 2005). A different motif, consisting solely of the bases CCAC, has been correlated with ABI4-dependent retrograde signaling, particularly when the motif is adjacent to, or overlaps with, a G-box motif (Koussevitzky et al., 2007). ABI4-inducible gene expression has also been shown to be dependent on sequences related to the S-box (CACCG) (Bossi et al., 2009, Reeves et al., 2011). In general, some genes participating in seed dormancy and germination as well as genes involved in non-seedling pathways have been documented as direct targets of ABI4.

ABI4 is involved in the regulation of primary seed dormancy by binding to CCAC and/or CACCG *cis* elements and directly repressing the expression of the ABA inactivating genes *CYP707A1* and *CYP707A2* to cause a decline in ABA degradation, while activating *GA2ox7* expression to promote GA degradation (Shu et al., 2013, Shu et al., 2016a). In addition, ABI4 also promotes the transcription of a flowering gene, *FLC*, by binding to the CACC elements in its promoter (Shu et al., 2016b). Furthermore, ABI4 mediates the transcriptional repression of some ripening-related ethylene biosynthesis genes such as *ACS4*, *ACO2*, and *ACS8* by binding directly to the CCAC elements present in their promoters, thus reducing ethylene levels (Dong et al., 2016). Interestingly, a recent report indicated that ABI4 is indispensable for repressing the expression of *ARR6*/*7*/*15*, which are involved in seed dormancy, by binding to the CE1 elements (CACCG) located in the gene promoters (Huang et al., 2017). ABI4 also represses the expression of a defense-related gene, *VTC2*, by binding to its promoter and thereby modulating the reactive oxygen species (ROS) levels in *Arabidopsis* (Yu et al., 2019). ABI4 positively regulates the expression of *YangYing1* (*YY1*), which encodes a zinc finger transcription factor and is involved in antagonizing ABR1 (Li et al., 2016b), and DPG1 (Delayed Pale Greening 1) (Yi et al., 2019) during salt and drought stress responses, respectively. In addition, it has been recently reported that ABI4 promotes *phytochrome A* (*PHYA*) expression during seed germination in *Arabidopsis* (Barros-Galvao et al., 2019).

The distinct functions of ABI4 in repression and activation of different target genes is an interesting phenomenon for future evaluation, as it raises the question of how a single transcription factor carries out two separate functions. The main reason for these diverse actions from our point of view is that ABI4 might interact with one or more other transcription factors or transcription co-factors to perform distinct biological functions. To answer the question of what drives the transition of ABI4 from a repressor to an activator, we propose to screen for ABI4-interacting proteins in specific plant tissues such as seeds, roots, leaves, hypocotyls, flowers, and others, diverging from the approach taken by previous studies, which focused on whole seedlings. Understanding the spatial composition of ABI4-interacting proteins in different organs might identify distinct tissue-specific functional proteins and deepen our understanding of ABI4 functions.

## Controversial Role of ABI4 in Retrograde Signaling

**Figure 1:**
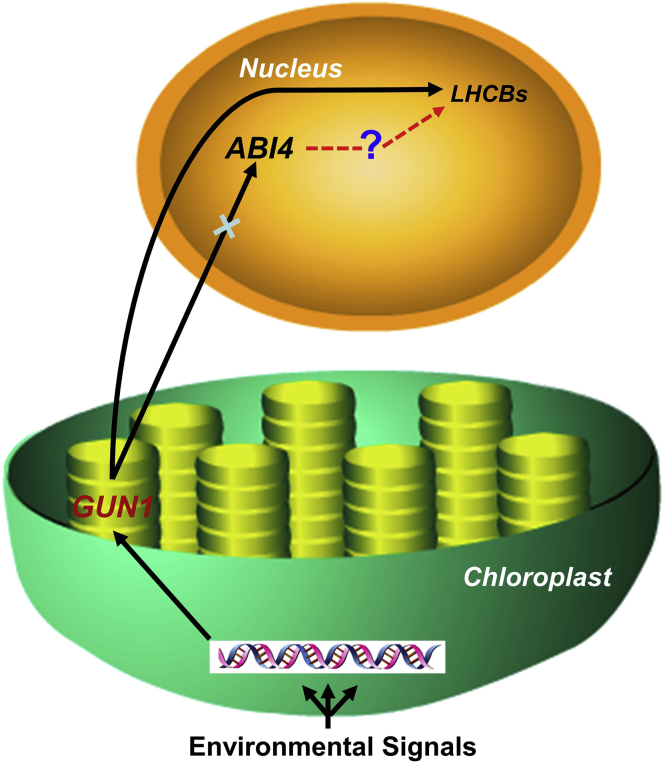
Controversial View on ABI4 Involvement in Plastid to Nucleus Retrograde Signaling. Recent evidence reported by Kacprzak et al. (2019) shows that ABI4 is not involved in retrograde signaling via *GUN1* as previously understood. Because the expression of *ABI4* increases under treatment with chloroplast inhibitors, we postulate the existence of an unknown signaling factor beyond *ABI4* that regulates expression of retrograde genes independently of *GUN1*; the unidentified factor is indicated by a dashed line.

It has been previously established that ABI4 represses the transcription of a plastid retrograde-regulated gene, LHCB1, through a conserved (CCAC) motif in its promoter (Koussevitzky et al., 2007). ABI4 also represses the transcript abundance of *AOX1a*, a mitochondrial retrograde signaling gene, by targeting to the CGTGAT elements in its promoter (Giraud et al., 2009). However, on a contrary note, a more recent report indicates that ABI4 is not required for GUN1-mediated plastid to nucleus retrograde signaling during chloroplast biogenesis (Kacprzak et al., 2019) (Figure 1). Kacprzak and colleagues stated that the expression of the chloroplast retrograde signaling genes *LHCBs* under treatment with chloroplast biogenesis inhibitors such as NF (norflurazon) or Lin (lincomycin) was not rescued in *abi4* as observed in *gun1*, signifying that ABI4 is not essential for chloroplast retrograde signaling. *ABI4* transcription was highly upregulated under NF or Lin treatments in wild type, but this induction was more strongly increased in *gun1* (Kacprzak et al., 2019), which is inconsistent with previous report (Sun et al., 2011). In addition to this, reanalysis of the transcriptome data previously obtained from *abi4* and *gun1* mutants indicated potential differences in the degree of overlap between ABI4- and GUN1-related genes (Koussevitzky et al., 2007, Martin et al., 2016, Kacprzak et al., 2019). From this finding, we speculate that there might be some other pathway by which ABI4 participates in the regulation of chloroplast biogenesis genes independently of GUN1, as indicated in Figure 1.

Proper nuclear localization is an essential step for uninterrupted functioning of many transcription factors, and in most cases is dependent on several interlinking proteins as well as other co-factors (Gregorio et al., 2014). Coordination of signals from the nucleus to other cellular organelles is extremely important, as misregulation of this event might lead to severe intercellular damage or create pseudo-signals. The effects of misregulation of ABI4 are evident from the work done by Gregorio et al. (2014) on an amino-terminal AP2-destabilizing domain truncation of ABI4, which resulted in overaccumulation of ABI4. Considering this example, a major study needs to be done on acetylation, phosphorylation, and disulfide bond formation, as well as other changes occurring during ABI4 nuclear localization. In addition, due attention should be paid in the coming years to exploring the unknown factors functioning in ABI4 signaling pathways independent of the GUN1 signaling pathway (Figure 1). The outcome of these proposed studies will be of great help to address the controversial omission of ABI4 from future nucleus retrograde signaling models as proposed by Kacprzak et al. (2019).

## Role of the ABI4 Protein Motif in Transregulation

ABI4 is known to bind to CACCG elements or CCAC elements for activation and repression of genes, but whether these different binding-site preferences result from specific protein modifications or from interaction with other proteins is currently unknown. Initially, deletion analysis studies showed that the sequences located within the first 224 amino acids were involved in the proteasome-mediated degradation of ABI4 (Finkelstein et al., 2011). Interestingly, these 224 amino acids were found to include a putative degradation motif (PEST) and a highly conserved 15-amino-acid sequence of the AP2 motif near the amino terminus of the protein (Finkelstein et al., 2011). It was concluded that several destabilizing domains might be involved in the degradation of ABI4 (Finkelstein et al., 2011).

In general, PEST sequences are involved in proteasome-mediated instability of different proteins (Finkelstein et al., 2011), but the exact role of this sequence in ABI4 protein stability is still not yet clear, although another report highlighted its predominant role in ABI4 degradation in a transient expression system (Gregorio et al., 2014). However, it is noteworthy that deletion of the AP2 domain resulted in the accumulation of ABI4 protein. Gregorio et al. (2014) also stated that the mechanisms behind PEST sequence recognition still needed substantial experimental confirmation. Thus, it will be intriguing to study PEST motif recognition site docking in future experiments, especially the role of the highly conserved serine (S) and threonine (T) residues in recognition.

Independent studies have identified various residues in the AP2/EREBP domain that participate in DNA binding of AP2 transcription factors (Liu et al., 2006). Several of these residues are highly conserved in the ABI4 proteins analyzed, suggesting that these residues might also be involved in DNA recognition in ABI4 proteins. Regrettably, experimental evidence for such recognition is very limited. A recent docking analysis using the AP2 domain of ABI4 predicted a binding structure similar to that of the *Arabidopsis* ERF1/AP2 domain (Wind et al., 2013). Supporting this, glycine 155 (G155), proline 164 (P164), and lysine 170 (K170) residues were found to be important for the ability of ABI4 to bind to the CE1-like motif (Wind et al., 2013). However, of these residues only K170 is specific to ABI4 compared with other DREBs and ERFs, suggesting it as a suitable candidate for conferring the DNA-binding specificity of ABI4 (Wind et al., 2013). On the other hand, other groups found that, in addition to the previously identified glutamic acid-69 residue, residues glutamine 151, serine 153, arginine 165, threonine 168, and K170 are highly conserved among ABI4 sequences but not among other DREB or ERF members (Laby et al., 2000, Wind et al., 2013, Gregorio et al., 2014), suggesting that these amino acids might determine the DNA-binding specificity of ABI4. However, no experimental evidence of such residue-binding activity is currently available. Thus, it will be important to uncover the possible residues involved in ABI4-binding specificity through experimental approaches in the future.

## Regulators of ABI4 at both the Transcript and Protein Levels

**Figure 2:**
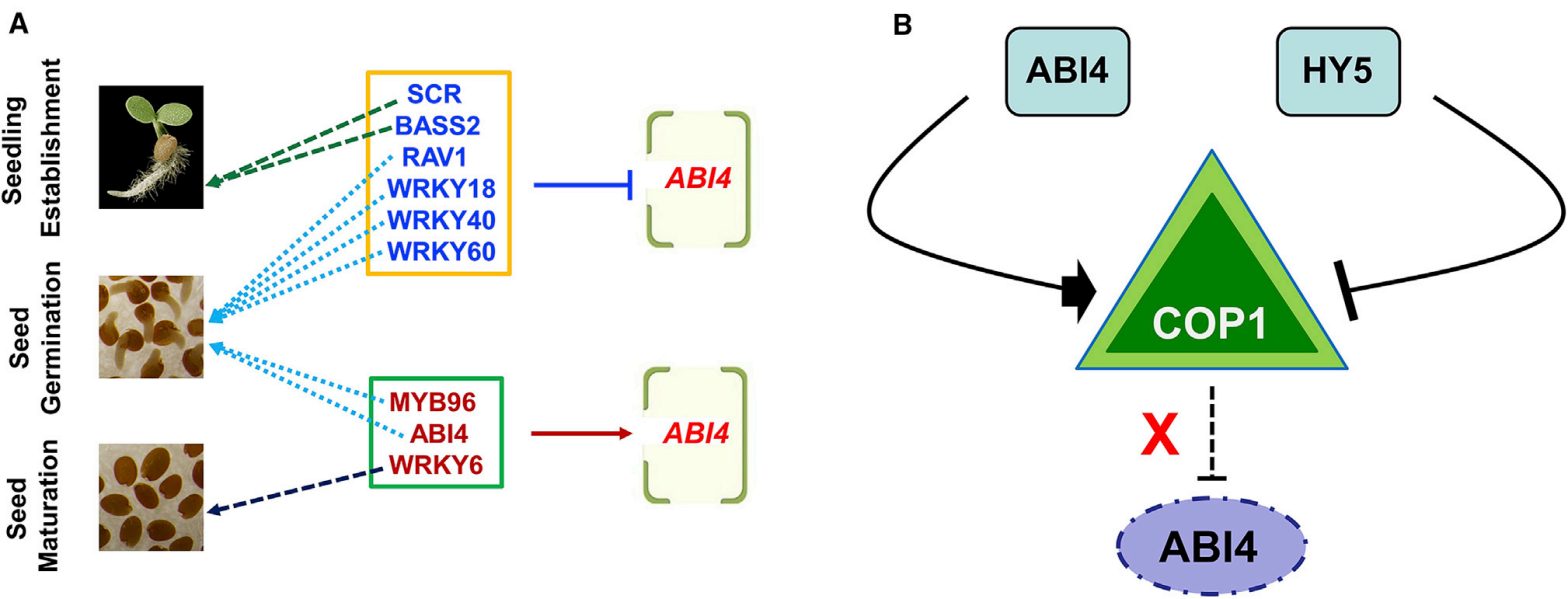
Transcriptional and Translational Regulators of ABI4. **(A)** Transcriptional level regulation involving transcription factors regulating *ABI4* transcript levels (positively and negatively) in different tissues or developmental stages. **(B)** The known factors involved in degradation of ABI4 protein; also highlighted is the unknown factor that is possibly directly involved in the ubiquitination of ABI4 and enhances its turnover. X indicates the unidentified subunit of E3 ligase directly participating in the degradation of the ABI4 protein.

The overall seedling development process requires integrated interactions between phytohormones and other environmental cues. Although ABI4 acts as a node of integration for different endogenous and exogenous signals in plants, limited evidence is available about its own regulatory mechanisms, both at the transcription and translation levels. The expression of *ABI4* is induced in the presence of low glucose (Cho et al., 2010) as well as high glucose and ABA (Arroyo et al., 2003, Zheng et al., 2019), and is also transiently induced by osmotic stress (Arroyo et al., 2003). Various transcription factors regulate *ABI4* transcription, including ABI4 itself, which is activated during the early stages of seedling growth (Bossi et al., 2009). Several WRKY transcription factors regulate *ABI4* expression by binding to the W-box sequence in its promoter. Specifically, WRKY6 promotes *ABI4* expression during seed maturation while the WRKY18/40/60 transcription factors repress *ABI4* transcription during seed germination (Shang et al., 2010, Antoni et al., 2011, Liu et al., 2012, Phukan et al., 2016, Ma et al., 2019). Furthermore, a GRAS domain family transcription factor, SCARECROW (SCR), modulates the sugar response in the root apical meristem by repressing *ABI4* expression upon binding directly to its promoter (Cui et al., 2012) (Figure 2A).

Transcription factor MYB96 promotes, while RAV1 and BASS2 repress, *ABI4* expression by binding to its promoter during seed germination and seedling development, respectively (Feng et al., 2014, Lee and Seo, 2015, Zhao et al., 2016) (Figure 2A). Intriguingly, Yellow Leaf 1 (YL1), a chloroplast-localized protein involved in plant salt stress response, indirectly represses *ABI4* expression (Li et al., 2016a). However, the intermediate factors connecting the gap between YL1 and ABI4 are currently elusive. Importantly, most of the regulators identified so far have been shown to repress *ABI4* expression while few have showed a promotional effect (Figure 2A). This evidence is consistent with the importance of negative regulation of *ABI4* level, as a higher level of ABI4 has been found to be harmful to seedlings because of the fact that ABI4 promotes ABA biosynthesis and represses GA biosynthesis (Shu et al., 2013, Shu et al., 2016a). Interestingly, despite its minimal expression in vegetative tissues, *ABI4* is expressed at high levels during seed maturation with expression decreasing during seed germination (Soderman et al., 2000, Shkolnik-Inbar and Bar-Zvi, 2011). In line with its roles in glucose signaling and regulating the expression of plastid proteins, expression of *ABI4* has been shown to increase dramatically in response to growth-inhibiting concentrations of glucose (Arroyo et al., 2003, Cho et al., 2010, Liu et al., 2018). Hence, it will be interesting to identify the transcriptional enhancers of *ABI4 in vivo*. Furthermore, since *ABI4* expression is controlled by diverse regulators such as MYB96, SCR, RAV1, BASS2, and WRKY 18/40/60, the regulatory mechanisms through which *ABI4* level is precisely controlled need to be clarified.

An earlier study showed that ABI4 is regulated post-transcriptionally, since the accumulation of its transcript does not correlate with its protein levels (Finkelstein et al., 2011). Intriguingly, transgenic plants overexpressing *ABI4* had undetectable levels of ABI4 protein despite high transcript accumulation, and in most cases the transgene was silenced after a few generations, suggesting that a high ABI4 protein level is harmful to plants (Finkelstein et al., 2011). The appropriate accumulation of ABI4 is a consequence of both post-transcriptional and post-translational regulation (Finkelstein et al., 2011, Gregorio et al., 2014). Initial studies using deletion analysis in transgenic plants showed that the sequences located within the first 224 amino acids are involved in the proteasome-mediated degradation of the ABI4 protein (Finkelstein et al., 2011).

Consequently, ABI4 is subjected to stringent post-transcriptional regulation that prevents the protein from accumulating at high level and restricts its action to a subset of tissues where its target genes are expressed (Finkelstein et al., 2011, Shu et al., 2016a). Protein modifications are known to affect the recognition of targets by the E3 ligases of the ubiquitin–26S proteasome system (Vierstra, 2009). Due to the unstable nature of the ABI4 protein (Finkelstein et al., 2011), the subunits of E3 ligases directly responsible for its degradation have been elusive so far. However, we propose to identify and mutate destabilizing domains that cause instability of ABI4, whereby the E3 ligases can probably be detected (Figure 2B). The barriers searching the responsible E3 ligases have been well documented previously (Shu and Yang, 2017, Kelley, 2018). In addition to this notion, chloroplast and light signals antagonistically fine-tune a suite of developmental and physiological responses associated with de-etiolation through a transcriptional module whereby ABI4 promotes and ELONGATED HYPOCOTYL 5 (HY5) inhibits the expression of *CONSTITUTIVE PHOTOMORPHOGENIC1* (*COP1*). In turn, ABI4 and HY5 are targeted for degradation by COP1 in light and dark conditions, respectively, to ensure proper crosstalk between ABI4 and HY5 during the seedling de-etiolation process (Xu et al., 2016) (Figure 2B). It is clear that COP1 promotes ABI4 degradation, as evidenced by *in vitro* pull-down and *in vivo* co-immunoprecipitation assays (Xu et al., 2016). However, the proteasomal degradation enzymes, especially the subunits of E3 ligases, involved in the degradation of ABI4 are currently unknown (Figure 2B). A focus on this question, especially on identifying the particular E3 ligase subunits, will reveal several key steps and enzymes participating in the turnover of the ABI4 protein.

## ABI4 Acts as a Cross-Mediator among Phytohormones or some Chemical Signals

Although ABI4 was first discovered with regard to its role in the ABA response during seed germination, numerous studies have reported it to be a highly versatile factor functioning in diverse chemical or phytohormone signaling pathways. Since the year 2010 there has been much important progress with regard to the roles of ABI4 in the crosstalk among several phytohormones. Here, we summarize the newly emerging evidence published in recent years.

### Positive Regulation of Jasmonic Acid and ROS Signaling by ABI4

**Figure 3:**
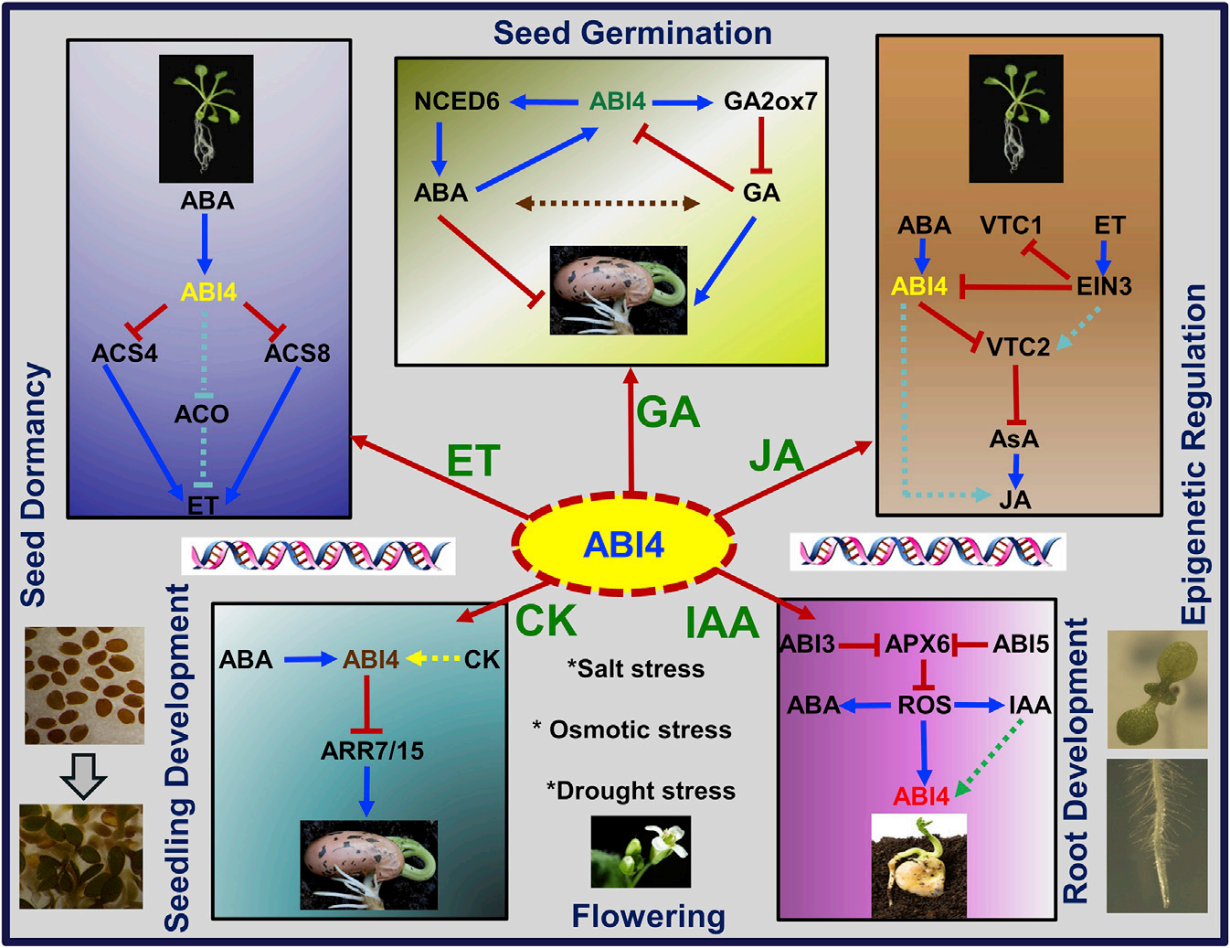
Model Illustrating Crosstalk between ABI4 and Phytohormones Involving the Direct Targets of Transcription Factor ABI4 Participating in Diverse Signaling Pathways. The *cis*-binding elements targeted by ABI4 are highlighted in black font. GA, unknown factors involved in ABI4-mediated gibberellic acid signaling are highlighted by a dashed line; JA, direct targets in the jasmonic signaling cascade that are targeted independently of ascorbate levels are highlighted; IAA, possible factors involved in auxin-mediated ABI4 signaling downstream of APX6 and ROS are proposed; ET, unidentified ethylene biosynthesis genes, in particular ACO enzymes, regulated by ABI4 are highlighted; CK, antagonistic effect of cytokinin on seed germination regulated by ABI4 is highlighted.

Ascorbate (AsA) plays crucial roles in photosynthesis and chloroplast functions and has been implicated in the control of the expression of genes encoding chloroplast proteins, similar to ABA. This function in turning on chloroplast proteins requires ABI4 activity (Kerchev et al., 2011). *abi4* reverses the slow growth phenotype as well as the altered gene expression patterns of the *vtc* mutant. Genetic analysis unequivocally demonstrates that the ascorbate-dependent regulation of plant growth requires ABI4. Thus, like other ABA signaling components, such as ABI1 and ABI2, which have long been known to function in stress signaling cascades involving ROS as second messengers, the evidence presented by Kerchev et al. (2011) also implicates ABI4 in redox signaling. In a more recent study, ethylene and ABA were found to co-regulate the ascorbate and ROS levels in seedlings, and *VTC2* was found to be the direct target of ABI4 in an *EIN3-ABI4-VTC2* signaling module (Yu et al., 2019). However, the factors participating in VTC2-directed AsA biosynthesis enforced by EIN3 are still unknown. This demonstrates that low ascorbate levels induced by ABA through the ABI4–VTC2 cascade activate ABA- and jasmonic acid-dependent signaling pathways that together regulate growth through the functional activation of ABI4 (Kerchev et al., 2011). In-depth analysis of this crosstalk mechanism will be important in further understanding the part of the ABI4 network that is independent of the ascorbate signaling pathway (Figure 3).

### Crosstalk between Auxin and ABA Involving ABI4

Auxin action in seed dormancy requires the ABA signaling pathway, indicating that the roles of auxin and ABA in seed dormancy are interdependent (Liu et al., 2013). High levels of auxin and activation of 3-indoleacetic acid (IAA) signaling enhance ABA-mediated dormancy by supporting the persistence of the expression of *ABI3* (Liu et al., 2013). Increased sensitivity of *apx6* (ascorbate peroxidase 6) mutant seeds to either ABA or IAA suggests that these hormones might be involved in the germination inhibition phenotype of the mutant (Chen et al., 2014). However, since *ABI3* and *ABI5* expression levels are relatively low in *apx6* seeds, it is likely that the crosstalk between auxin and ABA might also involve activation of other signaling pathways, preferentially the *ABI4* route (Figure 3). This can further be explained by the increased ROS level in *apx6*, which is associated with a higher expression level of *ABI4* in dry and imbibed seeds (Chen et al., 2014). Furthermore, crosstalk between ABA and auxin has also been demonstrated in *Arabidopsis* lateral root development (Shkolnik-Inbar and Bar-Zvi, 2010), primary root growth (Shkolnik-Inbar et al., 2013), and seed germination (Shkolnik-Inbar and Bar-Zvi, 2011, He et al., 2012). From this evidence, it is unclear whether ABI4 has a role in auxin signaling in these diverse plant developmental processes, and the role of ABI4 needs to be further explored in these as well as other plant parts, especially seed compartments.

### Negative Regulation of Ethylene Biosynthesis by ABI4

Gaseous ethylene is an important phytohormone involved in the regulation of plant development (e.g., floral organ development, fruit ripening, and senescence) and stress response (Kim et al., 2017, Li et al., 2017). ABA treatment prevents the induction of ethylene biosynthesis; this effect was tested using an ABA-deficient mutant of tomato, *aba2-1*, in which there was an increase in ethylene levels in shoots (Lenoble et al., 2003). Furthermore, the ABA-activated CDPK protein kinases CPK4 and CPK11 were found to stabilize ACS6 by phosphorylating its C terminus, promoting ethylene biosynthesis (Luo et al., 2014), and in another study ABI1, a negative regulator of ABA signaling, regulated ozone-induced ethylene biosynthesis by phosphorylating the C-terminal region of ACS6, which is controlled by MPK6 (Ludwikow et al., 2014). These findings reveal that the antagonistic interaction between ABA and ethylene is regulated by protein phosphorylation events. However, the mechanism of how ABA antagonizes ethylene biosynthesis at the transcriptional level remained unanswered until 2016, when Dong et al. (2016) found that a mutant harboring a dominant mutant allele of *ABI4*, *abi4-152*, which produced a putative protein with a 16-amino-acid truncation at the C terminus, had reduced ethylene production. By contrast, two mutants of *ABI4* with recessive knockout alleles, *abi4-102* and *abi4-103*, had increased ethylene production, indicating that ABI4 negatively regulates ethylene biosynthesis (Dong et al., 2016). This finding demonstrated that ABA negatively regulates ethylene production by repressing the expression of the major ethylene biosynthesis genes *ACS4* and *ACS8* by binding to their promoters (Dong et al., 2016). However, studies of the regulation of ethylene biosynthesis genes by ABI4 are very limited, and it will be interesting to discover more targets regulated by ABI4, especially other rate-limiting enzymes such as ACO (1-aminocyclopropane-1-carboxylic acid oxidase) and also downstream targets of the ethylene signaling pathway, as the need for ethylene synthesis varies among plant organs as well as in different plant species (Figure 3).

### ABI4 as a Modulator of Sugar-Sensing Signals

In plants, sugars function as signaling molecules that control important processes such as photosynthesis, growth, carbon distribution over different organs, and the production of storage compounds (Li et al., 2014). From previous studies it is known that the seedling growth and greening are inhibited under high concentrations of sugars, and that the sugar signaling pathways closely interact with other signaling pathways including phytohormone pathways (Zhu et al., 2009). Above all, ABA and sugar antagonistically form a complex cascade regulating thousands of genes involved in photosynthesis and metabolism (Yanagisawa et al., 2003, Dekkers et al., 2008). ABI4 is central regulator of sugar-responsive gene expression (Huijser et al., 2000, Laby et al., 2000). Recently, Li et al. (2014) identified a high-sugar super-sensitive line anac060, and found that ABI4 induces ANAC060 expression by interacting with its promoter and thereby rendering a glucose insensitive phenotype. Although previously reported studies provide some information on ABA -sugar crosstalk via ABI4, further identification of other critical genes regulated by ABI4 is urgently to understand ABA -sugar interaction.

### Antagonistic Role of ABI4 in Crosstalk between ABA and Gibberellins

Although studies of crosstalk between ABA and other phytohormones offer insight into key molecular mechanisms during seed development, the antagonism between ABA and gibberellin (GA) has been given much more attention, as GA is known to promote seed germination whereas ABA is known to inhibit seed germination (Shu et al., 2013, Shu et al., 2016a). Mutation of the *ABI4* locus completely rescues the non-germination phenotype of *ga1-1* mutant seeds, suggesting that ABI4 negatively regulates GA biosynthesis (Shu et al., 2013). Supporting this notion, in the *abi4* mutant ABA content is reduced whereas the GA content is increased, which opens up the possibility that ABI4 might play key roles in the antagonistic crosstalk between ABA and GA (Shu et al., 2013, Shu et al., 2016a). As with ABI4, a rice AP2 domain-containing transcription factor, *OsAP2-39*, is also involved in ABA/GA antagonism crosstalk (Yaish et al., 2010). *OsAP2-39* upregulates transcription of the ABA biosynthesis gene *OsNCED1* and leads to an increase in the endogenous ABA level. At the same time, *OsAP2-39* also enhances expression of the GA-inactivating gene *OsEUI* (*Elongated Uppermost Internode*), causing a decrease in endogenous GA content (Yaish et al., 2010). Taken together, these investigations have dissected only a portion of the novel mechanisms involved in the *in planta* control of the ABA/GA balance, which provides scope for further exploration of ABA and GA crosstalk (Figure 3).

### Negative Regulation of ABI4 in the Cytokinin Pathway

Cytokinin promotes seed germination and seedling establishment by antagonizing ABA signaling (Guan et al., 2014, Rowe et al., 2016, Verslues, 2016, Huang et al., 2018). Cytokinin signaling in general is transduced by a canonical two-component system involving a phosphorelay cascade (Huang et al., 2018). This cytokinin phosphorelay cascade contains histidine kinase receptors (AHKs), histidine phosphotransfer proteins (AHPs), and downstream response regulators (ARRs) (Huang et al., 2018). A recent study identified the role of ABA signaling in repressing *Arabidopsis ARR* genes, a class of cytokinin-inducible genes, and this repression of cytokinin-related genes by ABA was found to be mediated by ABI4 binding to their promoters (Huang et al., 2018). Genetic evidence shows that loss-of-function mutations of the *ARR7* and *ARR15* genes partially rescue the ABA insensitivity of the *abi4* mutant, revealing that ABI4 mediates ABA and cytokinin crosstalk by inhibiting the transcription of type-A *ARRs* during seed germination and cotyledon greening (Huang et al., 2017).

Taken together, the biological functions of other individual type-A ARRs during ABA response antagonizing with cytokinin and the regulatory relationship mediated by ABI4 require further exploration (Figure 3). It will be interesting to explore the interplay of ABI4 with cytokinin at various seed developmental stages by performing differential expression analysis (transcription as well as translation) under cytokinin treatment. The outcome of this type of study will define a genetic pathway integrating cytokinin signals and other co-factors mediated by ABI4. Future studies about their roles in pathways of other unexplored phytohormones will reveal their diverse capabilities.

## Concluding Remarks and Future Perspectives

The role of ABI4 in ABA signaling has been extensively studied in the past decades, whereas information about ABI4 beyond ABA is more attractive. Interestingly, several important research findings have been reported that ABI4 is multi-dimensional regulator. Subsequently, based on its functional roles in diverse signaling pathways, some key and unanswered questions have been pursued.

First of all, ABI4 participates in controlling the expression of a plethora of genes by operating as a positive and negative regulator; however, its modus operandi is currently unknown. How and why does this single transcription factor have two different functions? Thus, understanding the interacting proteins of ABI4 and their spatial structure should be of vital interest in the coming years.

Second, participation of ABI4 in retrograde signaling is unclear, as a recent report highlighted the fact that its role in chloroplast retrograde signaling is unsupported, although the expression of *ABI4* is induced under chloroplast inhibitor treatment. This opens up the possibility that chloroplast signaling genes are regulated independently of the GUN1 pathway. Further studies involving protein–protein interaction or transcriptome-wide analysis should be performed in an *abi4* mutant exposed to the chloroplast inhibitors NF and Lin to reveal unknown factors acting between ABI4 and *LHCB*s.

Third, although the mechanisms underlying crosstalk between ABA and other plant hormones are widely discussed, these discussions are on a basic level. For example, despite the antagonism between ABA and GA, ABI4 was found to rescue the non-germination phenotype of *ga1-t* mutants (Shu et al., 2013). The molecular mechanism behind this observation is currently unknown. Similarly, ABI4 negatively regulates major ethylene biosynthesis genes. However, the aspect of regulation of any ethylene-responsive factors by ABI4 that are located downstream of the ethylene signaling pathway is currently elusive. Also, ABI4 transduces signals from jasmonic acid in an indirect pathway dependent on AsA level, and any direct factors interacting with ABI4 are yet to be found. Similarly, direct involvement of cytokinin factors in regulation of ABI4 independently of the ARR signaling cascade needs experimental clarification. Furthermore, investigations of these questions are required to fully understand the mechanism by which ABI4 mediates crosstalk among phytohormones.

Fourth, high levels of ABI4 in seeds and vegetative tissues have been found to be harmful for plant development, so there exists a mechanism that modulates the ABI4 level for it to be under a threshold value. Although some transcriptional repressors have been found, the signaling pathways controlling ABI4 at the protein level need to be thoroughly assessed, especially the yet to be found subunits of E3 ligases. Do E3 ligases or any other regulators maintain ABI4 stability, considering that the instability of the ABI4 protein in nature is a major question to be answered? Detailed investigation of this degradation pathway and the involvement of phosphorylation, ubiquitination, sumoylation, or any other post-translational modification event is urgently needed to help identify the regulators functioning in modulating the ABI4 level in plant tissues.

Fifth, ABA-related phosphorylation mediated by SnRK2 kinases is required for ABI5 stability and activation as a transcription factor (Nakashima et al., 2009), but no such phosphorylation event occurring in ABI4 has yet been found. Identification of residues, such as glutamic acid 69, glutamine 151, serine 153, arginine 165, threonine 168, and lysine 170, in the ABI4 amino acid sequence might reveal phosphorylation site specificity (Gregorio et al., 2014). This initiates a quest for a detailed elucidation of any phosphorylation event occurring during the activation or repression of ABI4. In addition, although our understanding of ABI4 signaling has been clearly increased in the recent past, the connection of epigenetics to ABI4 action has not been thoroughly explored (Mu et al., 2017). Thus, it will also be intriguing to elucidate the molecular mechanisms behind epigenetic regulation of ABI4 in diverse plant organs.

Finally, the presence of the *ABI4* gene is vital, although its loss of function does not end a plant’s life unlike the loss of other genes such as Gibberellin Insensitive 1 (*GA1*) (Cao et al., 2005) or Albino Lethality 1 (*AL1*) (Zhang et al., 2016). As a signaling-responsive factor involved in pathways mediated by the plant stress hormone ABA, the essential role of *ABI4* in the plant life cycle is to modulate several critical genes to enable the plant to resist various abiotic stresses such as drought, salt, and osmotic conditions. For example, the *abi4* mutant is more resistant to high-salinity conditions compared with the wild-type seeds because it modulates the expression of *HKT* sodium transporter genes in *Arabidopsis* (Shkolnik-Inbar et al., 2013). In addition, the *HY1-ABI4-RbohD* complex maintains drought tolerance and plays a vital role in plant establishment under drought stress (Xie et al., 2016). Since plants are easily susceptible to environmental attacks, the frequent occurrence and participation of ABI4 in driving the expression of critical signaling genes involved in resisting environmental stress is substantial, and this clearly explains the functional centrality of ABI4 among diverse plant signaling networks. Altogether, shedding more light on the topics highlighted in this review will obtain many more outcomes to support the notion of ABI4 as a multifaceted transregulator.
